# The History and Development of HER2 Inhibitors

**DOI:** 10.3390/ph16101450

**Published:** 2023-10-12

**Authors:** Xiaohui Xia, Chen Gong, Yinan Zhang, Huihua Xiong

**Affiliations:** 1 Department of Oncology, Tongji Hospital, Tongji Medical College, Huazhong University of Science and Technology, Wuhan 430030, China; 2 School of Chemical Science and Engineering, Tongji University, Shanghai 200092, China

**Keywords:** HER2 inhibitors, cancer, antibodies, tyrosine kinase inhibitors, antibody-drug conjugates

## Abstract

HER2 is highly expressed in a variety of malignant tumors and affects the prognosis of patients, making it a highly sensitive target for cancer therapy. Since the approval of the first HER2 inhibitor, trastuzumab, in 1998, HER2-targeted drugs have rapidly evolved. Currently, targeting HER2 drugs mainly include monoclonal antibodies (mAbs), tyrosine kinase inhibitors (TKIs), and antibody-drug conjugates (ADCs). This article reviews the development of HER2 inhibitors for various tumors over the past 20 years.

## 1. Introduction

Cancer stands as a pressing global public health concern today. With the rapid advances in biotechnology, targeted therapy has surfaced as a promising treatment approach for cancer [1]. HER2, also known as ERBB2 and HER-2/neu, is a 185 kDa transmembrane protein encoded by the HER2 gene, which is situated on chromosome 17q21 [2]. HER2 belongs to the EGFR family of receptor tyrosine kinases and comprises three parts: extracellular domain (ECD), transmembrane domain (TMD), and intracellular domain (ICD) [3]. The extracellular domain can further be subdivided into four distinct subdomains (I–IV). The EGFR family includes HER 1, 2, 3, and 4. Unlike other members, HER2 lacks endogenous ligands and must form dimers with other members of the family to initiate downstream signaling pathways, such as the PI3K/Akt/mTOR and RAS-RAF-MEK-ERK pathway for cell growth and proliferation [4,5].

**Figure 1:**
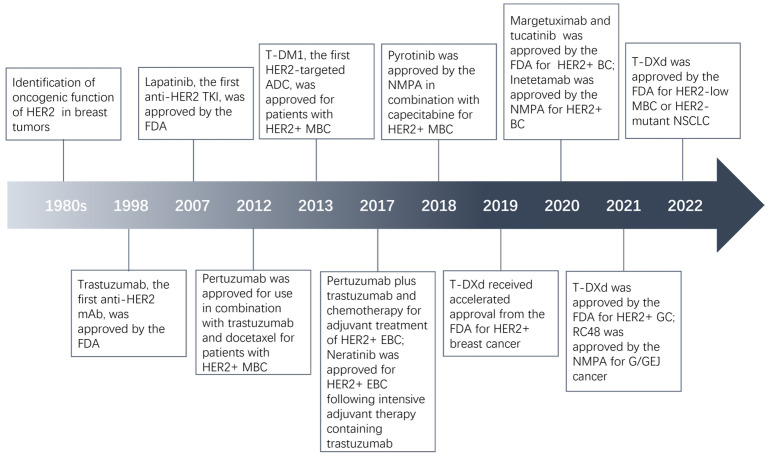
Major milestones in the evolution of HER2-targeted agents.

HER2 overexpression/amplification was found in many human malignant tumors, accounting for about 20% of breast and gastric cancer, 16% of epithelial ovarian cancer, and 1.3% of colorectal cancer [6,7,8,9,10]. In the 1980s, it was demonstrated to be a significant predictor of breast cancer [6]. Subsequently, this phenomenon was found in lung, ovarian, and gastric cancer [11,12,13]. Currently, HER2 has emerged as an important biomarker in the management of tumors, especially in breast cancer. The first monoclonal antibody (mAb) targeting HER2, trastuzumab, received approval from the Food and Drug Administration (FDA) back in 1998. It was approved for use as a monotherapy or in combination with paclitaxel to treat HER2-positive metastatic breast cancer (MBC) [14]. In 2007, lapatinib, the first anti-HER2 tyrosine kinase inhibitor (TKI), was used in patients with HER2-positive MBC in combination with capecitabine [15]. Since then, many HER2-targeted mAbs such as pertuzumab and margetuximab, and TKIs like neratinib and pyrotinib have been developed successfully and introduced into the clinic. Trastuzumab emtansine (T-DM1) belongs to antibody-drug conjugate (ADC), a class of drugs with a complex structure and special effects. In 2013, T-DM1, the first HER2-targeted ADC, was approved as a single agent by the FDA for patients with HER2-positive MBC who had been treated with trastuzumab and a taxane [16,17]. In 2019, another ADC, trastuzumab deruxtecan (T-DXd), was used for patients with HER2-positive breast cancer who had previously received HER2-targeted therapy [18]. Given the remarkable efficacy observed with trastuzumab and other medications in breast cancer, some HER2 inhibitors have been investigated for other tumors. For example, T-DXd received subsequent approvals for HER2-positive gastric cancer in 2021 and HER2-mutant non-small cell lung cancer (NSCLC) in 2022 [19,20]. Herein, we review the development of HER2 inhibitors in a variety of tumors over the past 20 years and focus on ADCs. Figure 1 shows the major milestones of HER2 inhibitors.

## 2. Antibodies

### 2.1. Trastuzumab (Herceptin)

Trastuzumab reduces tumor growth and survival by binding to the ECD subdomain IV of HER2, blocking downstream signaling pathways, and inducing antibody-dependent cellular cytotoxicity (ADCC) [21]. The original phase II trial demonstrated the safety and efficacy of trastuzumab in patients with HER2-positive MBC previously receiving extensive therapy [22]. In a phase III trial containing 469 patients with MBC and overexpression of HER2, patients received chemotherapy alone or in combination with trastuzumab [23]. Trastuzumab plus chemotherapy improved the median progression-free survival (PFS) by 2.8 months, median survival by 4.8 months, and objective response rate (ORR) by 18%. Cardiac dysfunction was the most significant adverse event (AE). Subsequently, NCT00004067 and NCT00005970 evaluated trastuzumab in patients with early-stage HER2-positive breast cancer. A total of 4046 patients with operable breast cancer who tested positive for the HER2 gene were enrolled in both studies and received doxorubicin plus cyclophosphamide, followed by paclitaxel with or without trastuzumab. Trastuzumab plus chemotherapy led to increased 10-year overall survival (OS) (84% vs. 75.2%) and 10-year PFS (73.7% vs. 62.2%) [24,25]. This great success has prompted trials of trastuzumab in other malignancies. In the landmark ToGA trial, trastuzumab combined with conventional chemotherapy improved OS by 2.7 months in HER2-positive advanced patients with gastric or gastro-oesophageal junction (G/GEJ) cancer compared with chemotherapy alone [26]. Trastuzumab plus chemotherapy then became the first-line treatment for patients with HER2-positive advanced gastric cancer.

### 2.2. Pertuzumab (Perjeta)

Pertuzumab, an IgG1 mAb targeting the ECD subdomain II of HER2, prevents HER2 from forming dimers [27]. In a phase III, randomized, multicenter, international clinical trial (NCT00567190), researchers assessed the effectiveness and safety of trastuzumab, combined with pertuzumab and chemotherapy, as the first-line treatment in 808 participants who were diagnosed with HER2-positive MBC. Patients in the pertuzumab group exhibited a notably extended median PFS (18.5 vs. 12.4 months), as well as improved 8-year OS rates (37% vs. 23%) and median OS (57.1 vs. 40.8 months) compared with patients receiving trastuzumab plus chemotherapy [28,29]. These findings provided a basis for the approval of pertuzumab. Then, in December 2017, the combination of pertuzumab, trastuzumab, and chemotherapy gained approval for post-operative treatment of HER2-positive early breast cancer (EBC) [30,31,32]. Trastuzumab plus pertuzumab is also being studied in tumors beyond breast cancer [33,34,35,36,37,38,39,40,41].

### 2.3. Margetuximab (Margenza, MGAH22)

Margetuximab is an anti-HER2 chimeric IgG1 mAb derived from the precursor to trastuzumab that also targets the subdomain IV of HER2. Its Fc domain was engineered to boost binding to CD16A while decreasing binding to CD32B in order to further enhance the antitumor activity on the basis of trastuzumab [42]. In 2020, based on the findings of SOPHIA (NCT02492711), margetuximab was approved for chemotherapy combination treatment in patients with HER2-positive MBC who had previously undergone anti-HER2 regimens [43,44]. In this randomized phase III trial, researchers found that margetuximab plus chemotherapy exhibited better PFS over trastuzumab plus chemotherapy (median, 5.8 vs. 4.9 months) [45]. However, patients in the two groups had similar overall OS [46]. The use of margetuximab in patients with HER2-positive breast cancer and different CD16A allele variants remains to be explored. MARGOT (NCT04425018) is recruiting patients with a low affinity CD16 germline genotype.

Margetuximab is also being studied for HER2-positive gastro-oesophageal adenocarcinoma. For instance, 95 participants were enrolled and treated with margetuximab plus pembrolizumab in a phase I/II single-group trial (NCT02689284). Among the response-evaluable population, the ORR, disease control rate (DCR), median PFS, and OS were 18.48%, 53%, 2.73 months, and 12.48 months, respectively [47]. These findings suggested that combining HER2 inhibitors and immune checkpoint inhibitors could result in a greater antitumor effect than either strategy used alone. MAHOGANY (NCT04082364) is exploring the use of margetuximab plus retifanlimab for such patients.

### 2.4. Inetetamab (Cipterbin)

Inetetamab, a novel mAb binding to the HER2 receptor ECD subdomain IV, is currently only available in China and has been approved in combination with vinorelbine for HER2-positive breast cancer patients who have undergone chemotherapy by China National Medical Products Administration (NMPA) in July 2020. Like margetuximab, the Fc domain of inetetamab was engineered to produce a stronger ADCC effect. Patients receiving inetetamab and vinorelbine had longer PFS (39.1 vs. 14.0 weeks), higher ORR (46.7% vs. 18.45%), and DCR (79.72% vs. 45.63%) than patients receiving vinorelbine alone in a phase III trial [48]. Some studies of inetetamab in various circumstances are ongoing: inetetamab for advanced breast cancer (ABC) in combination with PD-1 inhibitor (NCT05291910) or pyrotinib (NCT05856383, NCT05823623, and NCT04681911) or pertuzumab (NCT05749016), inetetamab in combination with pyrotinib in HER2 mutant or amplified advanced NSCLC (NCT05016544), among others.

### 2.5. Zanidatamab (ZW25)

Bispecific antibodies (bsAbs) feature two different binding domains that allow them to attach to two antigenic epitopes simultaneously [49]. With the unique concept and incomparable advantages of mAbs, bsAbs are considered to be the next generation antibody. In the last decade, nine bsAbs have gotten FDA approval, seven of which are indicated for tumors. ZW25 is an IgG1 bispecific, biparatopic antibody binding to the ECD II and ECD IV domain of HER2 and has greater antitumor activity than trastuzumab plus pertuzumab preclinically through multiple antitumor mechanisms such as sufficient saturation binding to tumor cells [50]. In a phase I trial [51], ZW25 was well tolerated, with no dose-limiting toxicities. Diarrhea was the most common AE, and no treatment-related serious AEs or deaths occurred. Overall, among 83 evaluable HER2-expressing patients, 37 patients had an OR (ORR 31%; 95% CI 27.0–49.0), including 8 patients with biliary tract cancer (38%), 10 patients with colorectal cancer (38%), and 13 patients with other cancer types (36%). In a phase IIb study [52], researchers found that ZW was well tolerated and that patients with HER2-positive biliary tract cancer benefited more than those with low HER2 expression, as reflected in a longer median PFS (5.5 vs. 1.9 months). ZW25 is being evaluated for other HER2-positive tumors, including breast cancer (NCT05035836), colorectal cancer (NCT03929666), and gastric cancers (NCT05152147).

### 2.6. Others

1E11, a HER2-targeted mAb binding to ECD subdomain IV, inhibited tumor growth when combined with trastuzumab in the preclinical model of gastric cancer [53]. KN026, a new bsAb based on trastuzumab and pertuzumab, exhibited comparable efficacy as the two drugs in patients with HER2-positive MBC [54]. Furthermore, KN026 demonstrated favorable safety and antitumor activity for patients with advanced G/GEJ cancer having high-level HER2 expression [55]. There are presently a dozen clinical trials of KN026 registered at ClinicalTrials.gov, primarily involving breast cancer and G/GEJ cancer. MBS301, another bsAb that has been glycoengineered from trastuzumab and patuzumab, is currently being studied in HER2-positive recurrent or metastatic tumors (NCT03842085) [56].

## 3. Tyrosine Kinase Inhibitors

### 3.1. Lapatinib (Tykerb)

Lapatinib is the first TKI targeting HER2 to be approved that reversibly competes with adenosine triphosphate for its binding site on the tyrosine kinase domain and inhibits downstream pathways and tumor cell proliferation [15]. Combination therapy (lapatinib plus capecitabine) was well tolerated and reduced disease progression events (49 vs. 72) and extended PFS (8.4 vs. 4.4 months) in a large phase III trial for HER2-positive patients with locally advanced or metastatic breast cancer, which established its initial approval in 2007 [57,58]. At present, lapatinib has only shown good antitumor efficacy in breast cancer, but studies in other tumors are still ongoing such as glioma (NCT02101905).

### 3.2. Neratinib (Nerlynx, HKI-272)

Neratinib is an irreversible, pan-HER TKI that inhibits the proliferation of tumor cells by reducing EGFR and HER2 autophosphorylation and inhibiting the downstream pathways including MAPK and AKT [59]. The ExteNET (NCT00878709) enrolled 2840 participants with HER2-positive EBC who had already undergone the standard duration of trastuzumab treatment. In this trial, neratinib as an extended adjuvant therapy reduced the invasive disease-free survival (iDFS) events, but meaningful improvement in OS was not observed [60,61]. TBCRC 022 showed good effects of combining neratinib with capecitabine for the treatment of HER2-positive breast cancer and central nervous system (CNS) metastases [62,63]. Cristina Saura et al. found that the combination treatment extended PFS and reduced the interventions for CNS disease compared to lapatinib plus capecitabine [64]. Based on these findings, the FDA granted approval for neratinib in July 2017 for HER2-positive patients with EBC who had undergone intensive adjuvant therapy containing trastuzumab, and it was also approved in combination with capecitabine in February 2020 for patients with advanced or metastatic HER2-positive breast cancer who have previously received two or more anti-HER2 regimens. Currently, neratinib in combination with ADCs is also being evaluated in MBC patients who have previously been treated with trastuzumab and pertuzumab-based therapies [65]. Neratinib is also being studied in colorectal cancer (NCT03457896).

### 3.3. Tucatinib

Tucatinib is a highly selective TKI targeting HER2 without significantly affecting EGFR [66]. Tucatinib has demonstrated therapeutic activity both as a single agent and in combination with chemotherapy or other HER2 inhibitors. In the HER2CLIMB trial, the use of tucatinib in combination with trastuzumab and capecitabine improved OS (21.6 vs. 12.5 months) in patients with HER2-positive MBC over those in the placebo-combination group with acceptable toxicity and also improved CNS-PFS (9.9 vs. 4.2 months) among the patients with brain metastases [67,68,69,70]. Based on these findings, tucatinib got the FDA approval in 2020 for the treatment of HER2-positive MBC, including brain metastatic. Furthermore, in the MOUNTAINEER trial, tucatinib was given with trastuzumab for patients with refractory HER2-positive metastatic colorectal cancer (mCRC). The trial reported an impressive ORR of 38% and demonstrated favorable safety profiles, which subsequently led to the FDA’s accelerated approval of tucatinib in January 2023 for this specific patient population [71,72].

### 3.4. Pyrotinib

Pyrotinib is an oral irreversible dual ErbB TKI developed by Jiangsu Hengrui Medicine and is applied and explored only in China at present. In August 2018, pyrotinib plus capecitabine was used for HER2-positive advanced or metastatic breast cancer [73]. Pyrotinib plus capecitabine showed activity and safety, with significant improvement in ORR and PFS when compared to the use of lapatinib plus capecitabine in patients with HER2-positive metastatic breast cancer [74,75,76]. NeoATP, a phase II clinical study, evaluated the effectiveness of pyrotinib in the neoadjuvant setting for EBC patients. The primary analysis demonstrated that patients treated with pyrotinib, trastuzumab, palitaxel, and cisplatin had a pathological complete response (pCR) rate of 69.81% [77]. This regime may be used in clinical practice in the future. Pyrotinib also exhibited antitumor activity in HER2-mutant NSCLC patients [78,79]. At present, many clinical studies are exploring pyrotinib in breast cancer and other solid tumors.

### 3.5. Poziotinib (HM781-36B)

Poziotinib is an irreversible pan-HER inhibitor that can bind in multiple sites of the HER family and inhibits downstream pathways [80]. Preclinical studies demonstrated its antitumor potency in HER2-amplified or HER2-mutant cancer models [81,82,83]. Poziotinib exhibited clinical benefit in HER2 exon 20-mutated NSCLC with an ORR of up to 39% with a manageable toxicity profile [84,85,86]. The main AEs reported were diarrhea and stomatitis. The NOV120101-203 trial showed the meaningful activity of poziotinib in patients with HER2-positive MBC, with a PFS of 4.04 months [87]. In a phase I/II trial of HER2-positive advanced gastric cancer, poziotinib in combination with paclitaxel and trastuzumab demonstrated a PFS of 13 weeks and an OS of 29.5 weeks [88]. Currently, poziotinib is mainly studied in HER2-mutant NSCLC. Although it is not approved by the FDA, it is still an important option for patients with NSCLC.

### 3.6. Others

Epertinib (S-222611) is a reversible and selective TKI of EGFR and HER2 that exhibited great antineoplastic activity over lapatinib in the preclinical study and is well tolerated in patients with solid tumors [89,90]. In a phase Ib trial, epertinib exhibited an ORR of 16% in HER2-positive breast cancer and 8.3% in upper gastrointestinal cancer [91]. Epertinib combined with trastuzumab also showed good effective antitumor activity in HER2-positive MBC patients who had previously undergone treatment [92]. DZD1516 is a highly selective HER2-target drug that possesses the ability to penetrate the blood–brain barrier. It is presently being evaluated in a phase I study (NCT04509596). JBJ-08-178-01 is synthesized to treat HER2-mutant tumors and has represented great growth inhibition in HER2 exon 20-mutated NSCLC models [93].

## 4. Antibody–Drug Conjugates

### 4.1. Trastuzumab Emtansine (T-DM1, Kadcyla)

ADCs are currently the fastest growing and promising class of antitumor drug that consist of three key elements: antibodies that target specific antigens, cytotoxic drug payloads, and linkers that can or cannot be cut [94]. Therefore, ADCs combine antibody cancer specificity with chemotherapeutic cytotoxicity and reduce toxic side effects on normal cells. A hundred years ago, it was proposed that there was a “magic bullet” drug that could specifically eliminate tumor cells through toxins and targeting agents. This idea became a reality eighty years later, with the first successful ADC. As of August 2023, there are two ADCs targeting HER2 approved by the FDA, one approved by the NPMA, one under review, and many more in clinical development. On 22 February 2013, T-DM1 developed by Genentech and ImmunoGen got the FDA approval for the indication of second-line monotherapy in patients with HER2-positive MBC with prior trastuzumab and taxane treatment, either individually or together [95]. Mertansine (DM1), a tubulin disruptor that inhibits mitosis and induces apoptosis, is linked to trastuzumab via an irreducible thioether linker. Every T-DM1 contains a trastuzumab molecule and an average of 3.6 DM1 molecules [96]. T-DM1 outperformed non-conjugated trastuzumab in preclinical models of HER2-positive breast, lung, and gastric cancer [96,97,98]. The first-in-human study of T-DM1 in HER2 breast cancer patients revealed a favorable tolerability profile and strong antineoplastic activity [99]. The landmark EMILIA trial enrolled 991 HER2-positive MBC participants who had previously been treated (NCT00829166). Patients randomly received T-DM1 or capecitabine and lapatinib. Researchers found that T-DM1 improved the PFS and OS relative to capecitabine plus lapatinib with fewer grade 3 or worse AEs [100,101]. These findings led to FDA approval of T-DM1 and other trials to test this ADC in MBC, EBC, and other HER2-expressing cancers. When used as the first-line treatment for patients with HER2-positive MBC, T-DM1 extended PFS to 14.2 months, surpassing the 9.2 months achieved with trastuzumab plus docetaxel, and maintained an acceptable safety profile [102]. In a randomized, multicenter, phase III trial including 1486 HER2-positive EBC patients (NCT01772472), adjuvant T-DM1 therapy was found to have a higher iDFS and fewer distant recurrence events than trastuzumab treatment [103]. Given the encouraging outcomes, T-DM1 received approval in 2019 for such patients. For patients with brain metastases, T-DM1 demonstrated a median PFS of 5.5 months and OS of 18.9 months, which warrants further exploration [104]. Combination therapy of T-DM1 with other agents is also being explored. T-DM1 plus pertuzumab or atezolizumab did not result in greater benefits to patients with ABC than T-DM1 alone [105,106]. T-DM1 plus pembrolizumab is being studied in patients with HER2-positive MBC who have prior treatment (NCT03032107) (Table 1). NCT02675829 and NCT02289833 displayed preliminary efficacy of T-DM1 in patients with HER2-overexpressing and HER2-mutant advanced lung cancer [107,108]. However, T-DM1 did not improve survival in patients with HER2-positive advanced gastric cancer [109].

### 4.2. Trastuzumab Deruxtecan (T-DXd, DS-8201, Enhertu)

T-DXd consists of three components: trastuzumab, a topoisomerase I inhibitor deruxtecan (DXd) that induces irreversible DNA damage and prevents DNA replication, and a novel cleavable linker with superior stability [110]. The molecular ratio of DXd to antibody is 7–8. T-DXd is the second approved ADC targeting HER2 after T-DM1 and has stronger effects and therapeutic potential due to its excellent structure. In December 2019, given the DESTINY-Breast01 (NCT03248492), the FDA gave fast track approval for T-DXd indicated for unresectable or metastatic HER2-positive breast cancer treated with HER2-targeted agents [111]. In the DESTINY-Breast03 trial (NCT03529110) comparing the effectiveness and safety of T-DXd and T-DM1 in HER2-positive patients with MBC, T-DXd exhibited substantially higher median PFS and OS than T-DM1, which reaffirmed T-DXd for second-line treatment [112]. Since T-DXd was first approved, numerous clinical studies have been conducted to explore the ADC in breast cancer with low HER2 expression and other tumors. In January 2021, the approval of T-DXd was granted for patients with locally advanced or metastatic HER2-positive gastric cancer who previously received a trastuzumab-based therapy as a result of the DESTINY-Gastric01 trial (NCT03329690) [19]. In August 2022, T-DXd was approved for the treatment of patients with HER2-low MBC or HER2-mutant NSCLC (NCT03734029, NCT03505710, and NCT04644237), becoming the first FDA-approved drug to treat HER2-mutant NSCLC. Furthermore, T-DXd is currently also being explored in colorectal cancer (NCT03384940), urothelial carcinoma (NCT03523572), and triple-negative breast cancer (NCT05953168). Table 1 showed some ongoing studies investigating the utilization of T-DXd in combination with other drugs. For instance, DESTINY-Breast09, which is recruiting patients, aims to evaluate the effectiveness and safety of T-DXd plus pertuzumab as a first-line treatment for HER2-positive MBC patients. Additionally, T-DXd in combination with pembrolizumab is also being explored in patients with locally advanced/metastatic breast cancer, as well as NSCLC.

### 4.3. Disitamab Vedotin (RC48, Aidixi)

RC48 is composed of a new HER2-targeted mAb hertuzumab, an antimitotic agent monomethyl auristatin E (MMAE), and a cleavable stable linker, exhibiting antitumor effects by inhibiting HER2 phosphorylation, inducing cell cycle arrest and apoptosis [113,114]. Preclinical studies and a phase I trial yielded good results in HER2-positive solid tumors [114,115]. RC48 was approved in June 2021 by NMPA for the treatment of patients with HER2-overexpressing locally advanced or metastatic G/GEJ cancer who have had more than one type of systemic chemotherapy regimens. In a phase II single-arm trial comprising 43 participants with HER2-overexpressing and refractory urothelial carcinoma (mUC), RC48 treatment revealed favorable outcomes with an ORR of 51.2%, a PFS of 6.9 months, and an OS of 13.9 months [116]. Another phase II trial evaluating RC48 in mUC also showed promising results [117]. In addition, RC48 is currently under investigation as a single agent or in combination therapy in NSCLC, urothelial carcinoma, breast cancer, and biliary tract cancer (Table 1).

### 4.4. Trastuzumab Duocarmazine (SYD985)

SYD985 consists of trastuzumab linked to a DNA alkylating agent (seco-DUBA) based on duocarmycins via a cleavable peptide linker [118]. SYD985 showed good antitumor activity in HER2-expressing breast, ovarian, and uterine cancer in vitro and in vivo [119,120,121,122]. The first-in-human trial (NCT02277717) found that SYD985 was well tolerated in refractory cancer patients with various HER2 statuses. The most common treatment-related severe AEs are infusion-related reactions and dyspnea. A promising efficacy was observed in patients with HER2-positive breast cancer (ORR 43%) and HER2-low breast cancer (ORR 28%) [123]. These study findings led to SYD985 receiving fast-track designation from the FDA. In a phase III trial (NCT03262935), 437 HER2-positive breast cancer patients were enrolled with 291 receiving SYD985 and 146 receiving physician’s choice. SYD985 showed a notably PFS improvement (median, 7 vs. 4.9 months). A single-arm phase II trial to assess the safety and efficacy of SYD985 as second-line therapy in endometrial cancer has been completed, but the results have not yet been reported (Table 1).

### 4.5. Others

A166 consists of trastuzumab, a microtubule inhibitor (duostatin-5), and a stable protease-cleavable valine citrulline linker. A phase I study demonstrated that A166 has manageable toxicity and promising antitumor activities in HER2-positive breast cancer patients [124]. A recently completed open-label, phase I-II study investigated A166 as a monotherapy for patients with solid tumors expressing or amplifying HER2, who progressed on or did not respond to available standard therapies (NCT03602079).

ARX-788, created by coupling the dolastatin analog, MMAF, to the HER2 antibody at two particular locations via a non-cleavable linker, showed antineoplastic effects in various solid tumors [125]. In phase 1 clinical trials, the ADC was well tolerated and had promising signs of activity in patients with HER2-positive MBC cancer and G/GEJ [126,127]. ACE-Breast-03, a global single-arm phase II trial, is testing ARX-788 in HER2-positive MBC patients who previously received T-DXd.

ALT-P7 (HM2-MMAE) is constituted of a trastuzumab variant and two MMAE molecules. A phase I study (NCT03281824) tested ALT-P7 in patients with HER2-positive MBC and showed dose-limiting toxicities [128].

BDC-1001 is a novel ADC composed of a trastuzumab biosimilar, immunostimulatory toll-like receptor (TLR) 7/8 agonist and a linker that cannot be cleaved. Therefore, the ADC has potential immunostimulating and antitumor activities. BDC-1001 is currently under investigation in a phase I/II clinical trial, both as a single agent and in combination with nivolumab, for patients with advanced HER2-expressing solid tumors (NCT04278144) [129].

PF-06804103 is composed of an anti-HER2 mAb, tubulin disruptor auristatin-0101, and a linker that is cleavable by proteases [130]. But in the phase I trial of PF-06804103, many AEs were observed, and nearly half of the patients discontinued treatment as a result [131].

MRG002 consists of modified trastuzumab, MMAE, and a cleavable linker. In a phase I trial, MRG002 displayed tolerable toxicity with antitumor effects in patients with HER2-positive advanced or metastatic solid tumors including breast, gastric, and other tumor types [132]. The phase II/III clinical trial evaluating MRG002 in MBC is ongoing (NCT04924699).

ZW49 is a bispecific ADC composed of ZW25 and proprietary auristatin toxin with a protease-cleavable linker that was considered a potential therapeutic candidate in HER2-expressing cancers [133]. The preliminary findings from a phase I clinical trial indicated that ZW49 had acceptable safety and encouraging single-agent antitumor activity (NCT03821233) [134].

GQ1001 is a HER2-targeted mAb covalently linked to a cytotoxin of DM1. The first-in-human study of this agent (NCT04450732) is recruiting HER2-positive patients with advanced solid tumors.

SBT6050 is a HER2-targeted mAb conjugated to a TLR 8 agonist. Preclinical studies reported that SBT6050 had robust single agent efficacy and resistance to tumor rechallenge [135], prompting the first-in-human study testing this ADC in combination with PD-1 inhibitors (NCT04460456).

## 5. Conclusions and Future Directions

The discovery of the HER2 target and the development of effective HER2 inhibitors are major breakthroughs in cancer therapy. HER2-targeted therapy has improved survival and prognosis in some cancer patients, no longer limited to breast cancer but gradually extending to various tumors such as gastric cancer and transitioning from advanced to early stages. In addition, the conduct of a large number of clinical trials provides patients with opportunities for treatment. However, HER2 inhibitors are currently facing several challenges. The efficacy in HER2-negative tumors is limited, and not all patients with HER2-positive solid tumors can benefit from HER2-targeted therapy. The effect in breast cancer cannot be fully replicated in other types of tumors, possibly because of intratumoral heterogeneity in HER2 status [136]. The rapid emergence of new drugs complicates combination drug regimes. Resistance and side effects are also a concern. Therefore, the universal standard for screening patients who can receive anti-HER2 therapy and more personalized treatment options is urgently needed. In addition, the search for more effective and safer drugs remains a major task today.

Over the past two decades, increasingly mature pharmaceutical and biotechnological technology has enabled the manufacture of medications with a fine and complex structure such as ADCs, which determine the therapeutic success. The right selection and combination of targets, antibodies, cytotoxic payloads, and linker with bioconjugation techniques is the key and difficult point, which defines ADC toxicities and effectiveness to a certain extent. HER2 is a very popular target of ADCs. As mentioned above, there are many HER2-targeted ADCs currently being explored as a single agent or in combination with other drugs in clinical trials that are involved in various types of tumors. In the next few years, it is expected that ADCs will obtain more approval and may even replace traditional chemotherapy in some indications.

## Figures and Tables

**Table 1:** Select current clinical trials of anti-HER2 ADCs.

Drug Name	Treatment	Condition and Disease	Type of Study	Sponsor	Clinical Trial
T-DM1	T-DM1 + pembrolizumab	HER2-positive MBC	Phase 1b, single group	Dana-Farber Cancer Institute, Boston, MA, USA	NCT03032107
T-DXd	T-DXd + pertuzumab	HER2-positive MBC	Phase 3, randomized	AstraZeneca, Cambridge, UK	NCT04784715
	T-DXd + pembrolizumab	Locally advanced/metastatic breast cancer or NSCLC	Phase 1b, 2-part, non-randomized, multiple-dose study	Daiichi Sankyo, Inc., Tokyo, Japan	NCT04042701
RC48	RC48	Advanced NSCLC with HER2 overexpression or HER2 mutation	Phase 1/2, single group	RemeGen Co., Ltd., Yantai, China	NCT04311034
	RC48 + pembrolizumab	Locally advanced unresectable or metastatic urothelial carcinoma	Phase 2, multicohort	Seagen Inc., Bothell, WA, USA	NCT04879329
	RC48	HER2-positive and HER2-low expressing MBC with abnormal activation of PAM pathway.	Phase 2, single group	Cancer Institute and Hospital, Chinese Academy of Medical Sciences, Beijing, China	NCT05331326
	RC48 + envafolimab	Locally advanced or metastatic HER2-positive biliary tract cancer	Phase 2, single group	Jiangsu Cancer Institute & Hospital, Nanjing, China	NCT05417230
SYD985	SYD985	Recurrent, advanced, or metastatic endometrial carcinoma	Phase 2, single group	Byondis B.V., Nijmegen, The Netherlands	NCT04205630
A166	A166	HER2-expressing or amplified solid tumors	Phase 1/2	Klus Pharma Inc., East Windsor, NJ, USA	NCT03602079
ARX-788	ARX-788	HER2-positive MBC	Phase 2, single group	Ambrx, Inc., La Jolla, CA, USA	NCT04829604
BDC-1001	BDC-1001 + nivolumab	HER2-expressing advanced malignancies	Phase 1/2	Bolt Biotherapeutics, Inc., Redwood City, CA, USA	NCT04278144
MRG002	MRG002	HER2-positive, unresectable locally advanced or metastatic breast cancer	Phase 2/3, randomized	Shanghai Miracogen Inc., Shanghai, China	NCT04924699
ZW49	ZW49	Locally advanced (unresectable) or metastatic HER2-expressing cancers	Phase 1, first-in-human	Zymeworks Inc., Vancouver, BC, Canada	NCT03821233
GQ1001	GQ1001	HER2-positive advanced solid tumors	Phase 1, first-in-human	GeneQuantum Healthcare (Suzhou) Co., Ltd., Suzhou, China	NCT04450732
SBT6050	SBT6050 and pembrolizumab/cemiplimab	HER2-positive solid tumors	Phase 1, 5-part	Silverback Therapeutics, Seattle, WA, USA	NCT04460456

MBC, metastatic breast cancer; NSCLC, non-small-cell lung cancer.

## Data Availability

Data sharing is not applicable.

## References

[1] Bedard P.L., Hyman D.M., Davids M.S., Siu L.L. (2020). Small molecules, big impact: 20 years of targeted therapy in oncology. Lancet.

[2] Rubin I., Yarden Y. (2001). The basic biology of HER2. Ann. Oncol..

[3] Cho H.S., Mason K., Ramyar K.X., Stanley A.M., Gabelli S.B., Denney D.W., Leahy D.J. (2003). Structure of the extracellular region of HER2 alone and in complex with the Herceptin Fab. Nature.

[4] Yarden Y., Sliwkowski M.X. (2001). Untangling the ErbB signalling network. Nat. Rev. Mol. Cell Biol..

[5] Zhang X.N., Gao Y., Zhang X.Y., Guo N.J., Hou W.Q., Wang S.W., Zheng Y.C., Wang N., Liu H.M., Wang B. (2023). Detailed curriculum vitae of HER2-targeted therapy. Pharmacol. Ther..

[6] Slamon D.J., Clark G.M., Wong S.G., Levin W.J., Ullrich A., McGuire W.L. (1987). Human breast cancer: Correlation of relapse and survival with amplification of the HER-2/neu oncogene. Science.

[7] Van Cutsem E., Bang Y.J., Feng-Yi F., Xu J.M., Lee K.W., Jiao S.C., Chong J.L., Lopez-Sanchez R.I., Price T., Gladkov O. (2015). HER2 screening data from ToGA: Targeting HER2 in gastric and gastroesophageal junction cancer. Gastric Cancer.

[8] Cloven N.G., Kyshtoobayeva A., Burger R.A., Yu I.R., Fruehauf J.P. (2004). In vitro chemoresistance and biomarker profiles are unique for histologic subtypes of epithelial ovarian cancer. Gynecol. Oncol..

[9] Consortium A.P.G. (2017). AACR Project GENIE: Powering Precision Medicine through an International Consortium. Cancer Discov..

[10] Ariga S. (2023). History and Future of HER2-Targeted Therapy for Advanced Gastric Cancer. J. Clin. Med..

[11] Andre F., Le Chevalier T., Soria J.C. (2004). Her2-neu: A target in lung cancer?. Ann. Oncol..

[12] Yu D., Wolf J.K., Scanlon M., Price J.E., Hung M.C. (1993). Enhanced c-erbB-2/neu expression in human ovarian cancer cells correlates with more severe malignancy that can be suppressed by E1A. Cancer Res..

[13] Gravalos C., Jimeno A. (2008). HER2 in gastric cancer: A new prognostic factor and a novel therapeutic target. Ann. Oncol..

[14] Baselga J., Norton L., Albanell J., Kim Y.M., Mendelsohn J. (1998). Recombinant humanized anti-HER2 antibody (Herceptin) enhances the antitumor activity of paclitaxel and doxorubicin against HER2/neu overexpressing human breast cancer xenografts. Cancer Res..

[15] Dhillon S., Wagstaff A.J. (2007). Lapatinib. Drugs.

[16] Casi G., Neri D. (2012). Antibody-drug conjugates: Basic concepts, examples and future perspectives. J. Control. Release.

[17] Ballantyne A., Dhillon S. (2013). Trastuzumab emtansine: First global approval. Drugs.

[18] Narayan P., Osgood C.L., Singh H., Chiu H.J., Ricks T.K., Chiu Yuen Chow E., Qiu J., Song P., Yu J., Namuswe F. (2021). FDA Approval Summary: Fam-Trastuzumab Deruxtecan-Nxki for the Treatment of Unresectable or Metastatic HER2-Positive Breast Cancer. Clin. Cancer Res..

[19] Shitara K., Bang Y.J., Iwasa S., Sugimoto N., Ryu M.H., Sakai D., Chung H.C., Kawakami H., Yabusaki H., Lee J. (2020). Trastuzumab Deruxtecan in Previously Treated HER2-Positive Gastric Cancer. N. Engl. J. Med..

[20] Li B.T., Smit E.F., Goto Y., Nakagawa K., Udagawa H., Mazières J., Nagasaka M., Bazhenova L., Saltos A.N., Felip E. (2022). Trastuzumab Deruxtecan in HER2-Mutant Non-Small-Cell Lung Cancer. N. Engl. J. Med..

[21] Hudis C.A. (2007). Trastuzumab--mechanism of action and use in clinical practice. N. Engl. J. Med..

[22] Baselga J., Tripathy D., Mendelsohn J., Baughman S., Benz C.C., Dantis L., Sklarin N.T., Seidman A.D., Hudis C.A., Moore J. (1996). Phase II study of weekly intravenous recombinant humanized anti-p185HER2 monoclonal antibody in patients with HER2/neu-overexpressing metastatic breast cancer. J. Clin. Oncol..

[23] Slamon D.J., Leyland-Jones B., Shak S., Fuchs H., Paton V., Bajamonde A., Fleming T., Eiermann W., Wolter J., Pegram M. (2001). Use of chemotherapy plus a monoclonal antibody against HER2 for metastatic breast cancer that overexpresses HER2. N. Engl. J. Med..

[24] Perez E.A., Romond E.H., Suman V.J., Jeong J.H., Sledge G., Geyer C.E., Martino S., Rastogi P., Gralow J., Swain S.M. (2014). Trastuzumab plus adjuvant chemotherapy for human epidermal growth factor receptor 2-positive breast cancer: Planned joint analysis of overall survival from NSABP B-31 and NCCTG N9831. J. Clin. Oncol..

[25] Romond E.H., Perez E.A., Bryant J., Suman V.J., Geyer C.E., Davidson N.E., Tan-Chiu E., Martino S., Paik S., Kaufman P.A. (2005). Trastuzumab plus adjuvant chemotherapy for operable HER2-positive breast cancer. N. Engl. J. Med..

[26] Bang Y.J., Van Cutsem E., Feyereislova A., Chung H.C., Shen L., Sawaki A., Lordick F., Ohtsu A., Omuro Y., Satoh T. (2010). Trastuzumab in combination with chemotherapy versus chemotherapy alone for treatment of HER2-positive advanced gastric or gastro-oesophageal junction cancer (ToGA): A phase 3, open-label, randomised controlled trial. Lancet.

[27] Metzger-Filho O., Winer E.P., Krop I. (2013). Pertuzumab: Optimizing HER2 blockade. Clin. Cancer Res..

[28] Baselga J., Cortés J., Kim S.B., Im S.A., Hegg R., Im Y.H., Roman L., Pedrini J.L., Pienkowski T., Knott A. (2012). Pertuzumab plus trastuzumab plus docetaxel for metastatic breast cancer. N. Engl. J. Med..

[29] Swain S.M., Miles D., Kim S.B., Im Y.H., Im S.A., Semiglazov V., Ciruelos E., Schneeweiss A., Loi S., Monturus E. (2020). Pertuzumab, trastuzumab, and docetaxel for HER2-positive metastatic breast cancer (CLEOPATRA): End-of-study results from a double-blind, randomised, placebo-controlled, phase 3 study. Lancet Oncol..

[30] Howie L.J., Scher N.S., Amiri-Kordestani L., Zhang L., King-Kallimanis B.L., Choudhry Y., Schroeder J., Goldberg K.B., Kluetz P.G., Ibrahim A. (2019). FDA Approval Summary: Pertuzumab for Adjuvant Treatment of HER2-Positive Early Breast Cancer. Clin. Cancer Res..

[31] von Minckwitz G., Procter M., de Azambuja E., Zardavas D., Benyunes M., Viale G., Suter T., Arahmani A., Rouchet N., Clark E. (2017). Adjuvant Pertuzumab and Trastuzumab in Early HER2-Positive Breast Cancer. N. Engl. J. Med..

[32] Piccart M., Procter M., Fumagalli D., de Azambuja E., Clark E., Ewer M.S., Restuccia E., Jerusalem G., Dent S., Reaby L. (2021). Adjuvant Pertuzumab and Trastuzumab in Early HER2-Positive Breast Cancer in the APHINITY Trial: 6 Years’ Follow-Up. J. Clin. Oncol..

[33] Javle M., Borad M.J., Azad N.S., Kurzrock R., Abou-Alfa G.K., George B., Hainsworth J., Meric-Bernstam F., Swanton C., Sweeney C.J. (2021). Pertuzumab and trastuzumab for HER2-positive, metastatic biliary tract cancer (MyPathway): A multicentre, open-label, phase 2a, multiple basket study. Lancet Oncol..

[34] Meric-Bernstam F., Hurwitz H., Raghav K.P.S., McWilliams R.R., Fakih M., VanderWalde A., Swanton C., Kurzrock R., Burris H., Sweeney C. (2019). Pertuzumab plus trastuzumab for HER2-amplified metastatic colorectal cancer (MyPathway): An updated report from a multicentre, open-label, phase 2a, multiple basket study. Lancet Oncol..

[35] Friedman C.F., Hainsworth J.D., Kurzrock R., Spigel D.R., Burris H.A., Sweeney C.J., Meric-Bernstam F., Wang Y., Levy J., Grindheim J. (2022). Atezolizumab Treatment of Tumors with High Tumor Mutational Burden from MyPathway, a Multicenter, Open-Label, Phase IIa Multiple Basket Study. Cancer Discov..

[36] Hainsworth J.D., Meric-Bernstam F., Swanton C., Hurwitz H., Spigel D.R., Sweeney C., Burris H., Bose R., Yoo B., Stein A. (2018). Targeted Therapy for Advanced Solid Tumors on the Basis of Molecular Profiles: Results From MyPathway, an Open-Label, Phase IIa Multiple Basket Study. J. Clin. Oncol..

[37] Kurzrock R., Bowles D.W., Kang H., Meric-Bernstam F., Hainsworth J., Spigel D.R., Bose R., Burris H., Sweeney C.J., Beattie M.S. (2020). Targeted therapy for advanced salivary gland carcinoma based on molecular profiling: Results from MyPathway, a phase IIa multiple basket study. Ann. Oncol..

[38] Liu T., Qin Y., Li J., Xu R., Xu J., Yang S., Qin S., Bai Y., Wu C., Mao Y. (2019). Pertuzumab in combination with trastuzumab and chemotherapy for Chinese patients with HER2-positive metastatic gastric or gastroesophageal junction cancer: A subpopulation analysis of the JACOB trial. Cancer Commun..

[39] Tabernero J., Hoff P.M., Shen L., Ohtsu A., Shah M.A., Cheng K., Song C., Wu H., Eng-Wong J., Kim K. (2018). Pertuzumab plus trastuzumab and chemotherapy for HER2-positive metastatic gastric or gastro-oesophageal junction cancer (JACOB): Final analysis of a double-blind, randomised, placebo-controlled phase 3 study. Lancet Oncol..

[40] Tabernero J., Hoff P.M., Shen L., Ohtsu A., Shah M.A., Siddiqui A., Heeson S., Kiermaier A., Macharia H., Restuccia E. (2023). Pertuzumab, trastuzumab, and chemotherapy in HER2-positive gastric/gastroesophageal junction cancer: End-of-study analysis of the JACOB phase III randomized clinical trial. Gastric Cancer.

[41] Stroes C.I., Schokker S., Creemers A., Molenaar R.J., Hulshof M., van der Woude S.O., Bennink R.J., Mathot R.A.A., Krishnadath K.K., Punt C.J.A. (2020). Phase II Feasibility and Biomarker Study of Neoadjuvant Trastuzumab and Pertuzumab With Chemoradiotherapy for Resectable Human Epidermal Growth Factor Receptor 2-Positive Esophageal Adenocarcinoma: TRAP Study. J. Clin. Oncol..

[42] Nordstrom J.L., Gorlatov S., Zhang W., Yang Y., Huang L., Burke S., Li H., Ciccarone V., Zhang T., Stavenhagen J. (2011). Anti-tumor activity and toxicokinetics analysis of MGAH22, an anti-HER2 monoclonal antibody with enhanced Fcgamma receptor binding properties. Breast Cancer Res..

[43] Royce M., Osgood C.L., Amatya A.K., Fiero M.H., Chang C.J.G., Ricks T.K., Shetty K.A., Kraft J., Qiu J., Song P. (2022). FDA Approval Summary: Margetuximab plus Chemotherapy for Advanced or Metastatic HER2-Positive Breast Cancer. Clin. Cancer Res..

[44] Markham A. (2021). Margetuximab: First Approval. Drugs.

[45] Rugo H.S., Im S.A., Cardoso F., Cortés J., Curigliano G., Musolino A., Pegram M.D., Wright G.S., Saura C., Escrivá-de-Romaní S. (2021). Efficacy of Margetuximab vs Trastuzumab in Patients With Pretreated ERBB2-Positive Advanced Breast Cancer: A Phase 3 Randomized Clinical Trial. JAMA Oncol..

[46] Rugo H.S., Im S.A., Cardoso F., Cortes J., Curigliano G., Musolino A., Pegram M.D., Bachelot T., Wright G.S., Saura C. (2023). Margetuximab Versus Trastuzumab in Patients With Previously Treated HER2-Positive Advanced Breast Cancer (SOPHIA): Final Overall Survival Results From a Randomized Phase 3 Trial. J. Clin. Oncol..

[47] Catenacci D.V.T., Kang Y.K., Park H., Uronis H.E., Lee K.W., Ng M.C.H., Enzinger P.C., Park S.H., Gold P.J., Lacy J. (2020). Margetuximab plus pembrolizumab in patients with previously treated, HER2-positive gastro-oesophageal adenocarcinoma (CP-MGAH22-05): A single-arm, phase 1b-2 trial. Lancet Oncol..

[48] Bian L., Xu B.H., Di L.J., Wang T., Wang X.J., Jiao S.C., Yang J.L., Tong Z.S., Liu J., Feng J.F. (2020). Phase Ⅲ randomized controlled, multicenter, prospective study of recombinant anti-HER2 humanized monoclonal antibody (Cipterbin) combined with vinorelbine in patients with HER2 positive metastatic breast cancer: The HOPES Study. Zhonghua Yi Xue Za Zhi.

[49] Brinkmann U., Kontermann R.E. (2021). Bispecific antibodies. Science.

[50] Weisser N.E., Sanches M., Escobar-Cabrera E., O’Toole J., Whalen E., Chan P.W.Y., Wickman G., Abraham L., Choi K., Harbourne B. (2023). An anti-HER2 biparatopic antibody that induces unique HER2 clustering and complement-dependent cytotoxicity. Nat. Commun..

[51] Meric-Bernstam F., Beeram M., Hamilton E., Oh D.Y., Hanna D.L., Kang Y.K., Elimova E., Chaves J., Goodwin R., Lee J. (2022). Zanidatamab, a novel bispecific antibody, for the treatment of locally advanced or metastatic HER2-expressing or HER2-amplified cancers: A phase 1, dose-escalation and expansion study. Lancet Oncol..

[52] Harding J.J., Fan J., Oh D.Y., Choi H.J., Kim J.W., Chang H.M., Bao L., Sun H.C., Macarulla T., Xie F. (2023). Zanidatamab for HER2-amplified, unresectable, locally advanced or metastatic biliary tract cancer (HERIZON-BTC-01): A multicentre, single-arm, phase 2b study. Lancet Oncol..

[53] Ko B.K., Lee S.Y., Lee Y.H., Hwang I.S., Persson H., Rockberg J., Borrebaeck C., Park D., Kim K.T., Uhlen M. (2015). Combination of novel HER2-targeting antibody 1E11 with trastuzumab shows synergistic antitumor activity in HER2-positive gastric cancer. Mol. Oncol..

[54] Zhang J., Ji D., Cai L., Yao H., Yan M., Wang X., Shen W., Du Y., Pang H., Lai X. (2022). First-in-human HER2-targeted Bispecific Antibody KN026 for the Treatment of Patients with HER2-positive Metastatic Breast Cancer: Results from a Phase I Study. Clin. Cancer Res..

[55] Xu J., Ying J., Liu R., Wu J., Ye F., Xu N., Zhang Y., Zhao R., Xiang X., Wang J. (2023). KN026 (anti-HER2 bispecific antibody) in patients with previously treated, advanced HER2-expressing gastric or gastroesophageal junction cancer. Eur. J. Cancer.

[56] Huang S., Li F., Liu H., Ye P., Fan X., Yuan X., Wu Z., Chen J., Jin C., Shen B. (2018). Structural and functional characterization of MBS301, an afucosylated bispecific anti-HER2 antibody. MAbs.

[57] Geyer C.E., Forster J., Lindquist D., Chan S., Romieu C.G., Pienkowski T., Jagiello-Gruszfeld A., Crown J., Chan A., Kaufman B. (2006). Lapatinib plus capecitabine for HER2-positive advanced breast cancer. N. Engl. J. Med..

[58] Moy B., Kirkpatrick P., Kar S., Goss P. (2007). Lapatinib. Nat. Rev. Drug Discov..

[59] Deeks E.D. (2017). Neratinib: First Global Approval. Drugs.

[60] Holmes F.A., Moy B., Delaloge S., Chia S.K.L., Ejlertsen B., Mansi J., Iwata H., Gnant M., Buyse M., Barrios C.H. (2023). Overall survival with neratinib after trastuzumab-based adjuvant therapy in HER2-positive breast cancer (ExteNET): A randomised, double-blind, placebo-controlled, phase 3 trial. Eur. J. Cancer.

[61] Martin M., Holmes F.A., Ejlertsen B., Delaloge S., Moy B., Iwata H., von Minckwitz G., Chia S.K.L., Mansi J., Barrios C.H. (2017). Neratinib after trastuzumab-based adjuvant therapy in HER2-positive breast cancer (ExteNET): 5-year analysis of a randomised, double-blind, placebo-controlled, phase 3 trial. Lancet Oncol..

[62] Freedman R.A., Gelman R.S., Anders C.K., Melisko M.E., Parsons H.A., Cropp A.M., Silvestri K., Cotter C.M., Componeschi K.P., Marte J.M. (2019). TBCRC 022: A Phase II Trial of Neratinib and Capecitabine for Patients With Human Epidermal Growth Factor Receptor 2-Positive Breast Cancer and Brain Metastases. J. Clin. Oncol..

[63] Freedman R.A., Gelman R.S., Wefel J.S., Melisko M.E., Hess K.R., Connolly R.M., Van Poznak C.H., Niravath P.A., Puhalla S.L., Ibrahim N. (2016). Translational Breast Cancer Research Consortium (TBCRC) 022: A Phase II Trial of Neratinib for Patients With Human Epidermal Growth Factor Receptor 2-Positive Breast Cancer and Brain Metastases. J. Clin. Oncol..

[64] Saura C., Oliveira M., Feng Y.H., Dai M.S., Chen S.W., Hurvitz S.A., Kim S.B., Moy B., Delaloge S., Gradishar W. (2020). Neratinib Plus Capecitabine Versus Lapatinib Plus Capecitabine in HER2-Positive Metastatic Breast Cancer Previously Treated With >/= 2 HER2-Directed Regimens: Phase III NALA Trial. J. Clin. Oncol..

[65] Abraham J., Montero A.J., Jankowitz R.C., Salkeni M.A., Beumer J.H., Kiesel B.F., Piette F., Adamson L.M., Nagy R.J., Lanman R.B. (2019). Safety and Efficacy of T-DM1 Plus Neratinib in Patients With Metastatic HER2-Positive Breast Cancer: NSABP Foundation Trial FB-10. J. Clin. Oncol..

[66] Lee A. (2020). Tucatinib: First Approval. Drugs.

[67] Murthy R., Borges V.F., Conlin A., Chaves J., Chamberlain M., Gray T., Vo A., Hamilton E. (2018). Tucatinib with capecitabine and trastuzumab in advanced HER2-positive metastatic breast cancer with and without brain metastases: A non-randomised, open-label, phase 1b study. Lancet Oncol..

[68] Murthy R.K., Loi S., Okines A., Paplomata E., Hamilton E., Hurvitz S.A., Lin N.U., Borges V., Abramson V., Anders C. (2020). Tucatinib, Trastuzumab, and Capecitabine for HER2-Positive Metastatic Breast Cancer. N. Engl. J. Med..

[69] Lin N.U., Borges V., Anders C., Murthy R.K., Paplomata E., Hamilton E., Hurvitz S., Loi S., Okines A., Abramson V. (2020). Intracranial Efficacy and Survival With Tucatinib Plus Trastuzumab and Capecitabine for Previously Treated HER2-Positive Breast Cancer With Brain Metastases in the HER2CLIMB Trial. J. Clin. Oncol..

[70] Lin N.U., Murthy R.K., Abramson V., Anders C., Bachelot T., Bedard P.L., Borges V., Cameron D., Carey L.A., Chien A.J. (2023). Tucatinib vs Placebo, Both in Combination with Trastuzumab and Capecitabine, for Previously Treated ERBB2 (HER2)-Positive Metastatic Breast Cancer in Patients with Brain Metastases: Updated Exploratory Analysis of the HER2CLIMB Randomized Clinical Trial. JAMA Oncol..

[71] Casak S.J., Horiba M.N., Yuan M., Cheng J., Lemery S.J., Shen Y.L., Fu W., Moore J.N., Li Y., Bi Y. (2023). FDA Approval Summary: Tucatinib with trastuzumab for advanced unresectable or metastatic, chemotherapy refractory, HER2 positive RAS wild type colorectal cancer. Clin. Cancer Res..

[72] Strickler J.H., Cercek A., Siena S., André T., Ng K., Van Cutsem E., Wu C., Paulson A.S., Hubbard J.M., Coveler A.L. (2023). Tucatinib plus trastuzumab for chemotherapy-refractory, HER2-positive, RAS wild-type unresectable or metastatic colorectal cancer (MOUNTAINEER): A multicentre, open-label, phase 2 study. Lancet Oncol..

[73] Blair H.A. (2018). Pyrotinib: First Global Approval. Drugs.

[74] Ma F., Ouyang Q., Li W., Jiang Z., Tong Z., Liu Y., Li H., Yu S., Feng J., Wang S. (2019). Pyrotinib or Lapatinib Combined With Capecitabine in HER2-Positive Metastatic Breast Cancer With Prior Taxanes, Anthracyclines, and/or Trastuzumab: A Randomized, Phase II Study. J. Clin. Oncol..

[75] Xu B., Yan M., Ma F., Hu X., Feng J., Ouyang Q., Tong Z., Li H., Zhang Q., Sun T. (2021). Pyrotinib plus capecitabine versus lapatinib plus capecitabine for the treatment of HER2-positive metastatic breast cancer (PHOEBE): A multicentre, open-label, randomised, controlled, phase 3 trial. Lancet Oncol..

[76] Yan M., Ouyang Q., Sun T., Niu L., Yang J., Li L., Song Y., Hao C., Chen Z., Orlandi A. (2022). Pyrotinib plus capecitabine for patients with human epidermal growth factor receptor 2-positive breast cancer and brain metastases (PERMEATE): A multicentre, single-arm, two-cohort, phase 2 trial. Lancet Oncol..

[77] Yin W., Wang Y., Wu Z., Ye Y., Zhou L., Xu S., Lin Y., Du Y., Yan T., Yang F. (2022). Neoadjuvant Trastuzumab and Pyrotinib for Locally Advanced HER2-Positive Breast Cancer (NeoATP): Primary Analysis of a Phase II Study. Clin. Cancer Res..

[78] Wang Y., Jiang T., Qin Z., Jiang J., Wang Q., Yang S., Rivard C., Gao G., Ng T.L., Tu M.M. (2019). HER2 exon 20 insertions in non-small-cell lung cancer are sensitive to the irreversible pan-HER receptor tyrosine kinase inhibitor pyrotinib. Ann. Oncol..

[79] Zhou C., Li X., Wang Q., Gao G., Zhang Y., Chen J., Shu Y., Hu Y., Fan Y., Fang J. (2020). Pyrotinib in HER2-Mutant Advanced Lung Adenocarcinoma After Platinum-Based Chemotherapy: A Multicenter, Open-Label, Single-Arm, Phase II Study. J. Clin. Oncol..

[80] Borm F.J., Smit E.F. (2023). Poziotinib for HER2 Exon 20-Mutated NSCLC: Addition or Burden to the Therapeutic Arsenal?. J. Thorac. Oncol..

[81] Cha M.Y., Lee K.O., Kim M., Song J.Y., Lee K.H., Park J., Chae Y.J., Kim Y.H., Suh K.H., Lee G.S. (2012). Antitumor activity of HM781-36B, a highly effective pan-HER inhibitor in erlotinib-resistant NSCLC and other EGFR-dependent cancer models. Int. J. Cancer.

[82] Nam H.J., Kim H.P., Yoon Y.K., Hur H.S., Song S.H., Kim M.S., Lee G.S., Han S.W., Im S.A., Kim T.Y. (2011). Antitumor activity of HM781-36B, an irreversible Pan-HER inhibitor, alone or in combination with cytotoxic chemotherapeutic agents in gastric cancer. Cancer Lett..

[83] Robichaux J.P., Elamin Y.Y., Tan Z., Carter B.W., Zhang S., Liu S., Li S., Chen T., Poteete A., Estrada-Bernal A. (2018). Mechanisms and clinical activity of an EGFR and HER2 exon 20-selective kinase inhibitor in non-small cell lung cancer. Nat. Med..

[84] Elamin Y.Y., Robichaux J.P., Carter B.W., Altan M., Gibbons D.L., Fossella F.V., Lam V.K., Patel A.B., Negrao M.V., Le X. (2022). Poziotinib for Patients With HER2 Exon 20 Mutant Non-Small-Cell Lung Cancer: Results From a Phase II Trial. J. Clin. Oncol..

[85] Le X., Cornelissen R., Garassino M., Clarke J.M., Tchekmedyian N., Goldman J.W., Leu S.Y., Bhat G., Lebel F., Heymach J.V. (2022). Poziotinib in Non-Small-Cell Lung Cancer Harboring HER2 Exon 20 Insertion Mutations After Prior Therapies: ZENITH20-2 Trial. J. Clin. Oncol..

[86] Cornelissen R., Prelaj A., Sun S., Baik C., Wollner M., Haura E.B., Mamdani H., Riess J.W., Cappuzzo F., Garassino M.C. (2023). Poziotinib in Treatment-Naive NSCLC Harboring HER2 Exon 20 Mutations: ZENITH20-4, A Multicenter, Multicohort, Open-Label, Phase 2 Trial (Cohort 4). J. Thorac. Oncol..

[87] Park Y.H., Lee K.H., Sohn J.H., Lee K.S., Jung K.H., Kim J.H., Lee K.H., Ahn J.S., Kim T.Y., Kim G.M. (2018). A phase II trial of the pan-HER inhibitor poziotinib, in patients with HER2-positive metastatic breast cancer who had received at least two prior HER2-directed regimens: Results of the NOV120101-203 trial. Int. J. Cancer.

[88] Kim T.Y., Han H.S., Lee K.W., Zang D.Y., Rha S.Y., Park Y.I., Kim J.S., Lee K.H., Park S.H., Song E.K. (2019). A phase I/II study of poziotinib combined with paclitaxel and trastuzumab in patients with HER2-positive advanced gastric cancer. Gastric Cancer.

[89] Tanaka H., Hirata M., Shinonome S., Wada T., Iguchi M., Dohi K., Inoue M., Ishioka Y., Hojo K., Yamada T. (2014). Preclinical antitumor activity of S-222611, an oral reversible tyrosine kinase inhibitor of epidermal growth factor receptor and human epidermal growth factor receptor 2. Cancer Sci..

[90] Spicer J., Baird R., Suder A., Cresti N., Corbacho J.G., Hogarth L., Frenkel E., Matsumoto S., Kawabata I., Donaldson K. (2015). Phase 1 dose-escalation study of S-222611, an oral reversible dual tyrosine kinase inhibitor of EGFR and HER2, in patients with solid tumours. Eur. J. Cancer.

[91] Arkenau H.T., Italiano A., Mak G., Toulmonde M., Baird R.D., Garcia-Corbacho J., Plummer R., Flynn M., Forster M., Wilson R.H. (2018). An extended phase Ib study of epertinib, an orally active reversible dual EGFR/HER2 tyrosine kinase inhibitor, in patients with solid tumours. Eur. J. Cancer.

[92] Macpherson I.R., Spiliopoulou P., Rafii S., Saggese M., Baird R.D., Garcia-Corbacho J., Italiano A., Bonneterre J., Campone M., Cresti N. (2019). A phase I/II study of epertinib plus trastuzumab with or without chemotherapy in patients with HER2-positive metastatic breast cancer. Breast Cancer Res..

[93] Son J., Jang J., Beyett T.S., Eum Y., Haikala H.M., Verano A., Lin M., Hatcher J.M., Kwiatkowski N.P., Eser P.O. (2022). A Novel HER2-Selective Kinase Inhibitor Is Effective in HER2 Mutant and Amplified Non-Small Cell Lung Cancer. Cancer Res..

[94] Joubert N., Beck A., Dumontet C., Denevault-Sabourin C. (2020). Antibody-Drug Conjugates: The Last Decade. Pharmaceuticals.

[95] Amiri-Kordestani L., Blumenthal G.M., Xu Q.C., Zhang L., Tang S.W., Ha L., Weinberg W.C., Chi B., Candau-Chacon R., Hughes P. (2014). FDA approval: Ado-trastuzumab emtansine for the treatment of patients with HER2-positive metastatic breast cancer. Clin. Cancer Res..

[96] Lewis Phillips G.D., Li G., Dugger D.L., Crocker L.M., Parsons K.L., Mai E., Blattler W.A., Lambert J.M., Chari R.V., Lutz R.J. (2008). Targeting HER2-positive breast cancer with trastuzumab-DM1, an antibody-cytotoxic drug conjugate. Cancer Res..

[97] Barok M., Tanner M., Koninki K., Isola J. (2011). Trastuzumab-DM1 is highly effective in preclinical models of HER2-positive gastric cancer. Cancer Lett..

[98] Phillips G.D., Fields C.T., Li G., Dowbenko D., Schaefer G., Miller K., Andre F., Burris H.A., Albain K.S., Harbeck N. (2014). Dual targeting of HER2-positive cancer with trastuzumab emtansine and pertuzumab: Critical role for neuregulin blockade in antitumor response to combination therapy. Clin. Cancer Res..

[99] Krop I.E., Beeram M., Modi S., Jones S.F., Holden S.N., Yu W., Girish S., Tibbitts J., Yi J.H., Sliwkowski M.X. (2010). Phase I study of trastuzumab-DM1, an HER2 antibody-drug conjugate, given every 3 weeks to patients with HER2-positive metastatic breast cancer. J. Clin. Oncol..

[100] Verma S., Miles D., Gianni L., Krop I.E., Welslau M., Baselga J., Pegram M., Oh D.Y., Dieras V., Guardino E. (2012). Trastuzumab emtansine for HER2-positive advanced breast cancer. N. Engl. J. Med..

[101] Dieras V., Miles D., Verma S., Pegram M., Welslau M., Baselga J., Krop I.E., Blackwell K., Hoersch S., Xu J. (2017). Trastuzumab emtansine versus capecitabine plus lapatinib in patients with previously treated HER2-positive advanced breast cancer (EMILIA): A descriptive analysis of final overall survival results from a randomised, open-label, phase 3 trial. Lancet Oncol..

[102] Hurvitz S.A., Dirix L., Kocsis J., Bianchi G.V., Lu J., Vinholes J., Guardino E., Song C., Tong B., Ng V. (2013). Phase II randomized study of trastuzumab emtansine versus trastuzumab plus docetaxel in patients with human epidermal growth factor receptor 2-positive metastatic breast cancer. J. Clin. Oncol..

[103] von Minckwitz G., Huang C.S., Mano M.S., Loibl S., Mamounas E.P., Untch M., Wolmark N., Rastogi P., Schneeweiss A., Redondo A. (2019). Trastuzumab Emtansine for Residual Invasive HER2-Positive Breast Cancer. N. Engl. J. Med..

[104] Montemurro F., Delaloge S., Barrios C.H., Wuerstlein R., Anton A., Brain E., Hatschek T., Kelly C.M., Pena-Murillo C., Yilmaz M. (2020). Trastuzumab emtansine (T-DM1) in patients with HER2-positive metastatic breast cancer and brain metastases: Exploratory final analysis of cohort 1 from KAMILLA, a single-arm phase IIIb clinical trial. Ann. Oncol..

[105] Perez E.A., Barrios C., Eiermann W., Toi M., Im Y.H., Conte P., Martin M., Pienkowski T., Pivot X.B., Burris H.A. (2019). Trastuzumab emtansine with or without pertuzumab versus trastuzumab with taxane for human epidermal growth factor receptor 2-positive advanced breast cancer: Final results from MARIANNE. Cancer.

[106] Emens L.A., Esteva F.J., Beresford M., Saura C., De Laurentiis M., Kim S.B., Im S.A., Wang Y., Salgado R., Mani A. (2020). Trastuzumab emtansine plus atezolizumab versus trastuzumab emtansine plus placebo in previously treated, HER2-positive advanced breast cancer (KATE2): A phase 2, multicentre, randomised, double-blind trial. Lancet Oncol..

[107] Li B.T., Shen R., Buonocore D., Olah Z.T., Ni A., Ginsberg M.S., Ulaner G.A., Offin M., Feldman D., Hembrough T. (2018). Ado-Trastuzumab Emtansine for Patients With HER2-Mutant Lung Cancers: Results From a Phase II Basket Trial. J. Clin. Oncol..

[108] Peters S., Stahel R., Bubendorf L., Bonomi P., Villegas A., Kowalski D.M., Baik C.S., Isla D., Carpeno J.C., Garrido P. (2019). Trastuzumab Emtansine (T-DM1) in Patients with Previously Treated HER2-Overexpressing Metastatic Non-Small Cell Lung Cancer: Efficacy, Safety, and Biomarkers. Clin. Cancer Res..

[109] Thuss-Patience P.C., Shah M.A., Ohtsu A., Van Cutsem E., Ajani J.A., Castro H., Mansoor W., Chung H.C., Bodoky G., Shitara K. (2017). Trastuzumab emtansine versus taxane use for previously treated HER2-positive locally advanced or metastatic gastric or gastro-oesophageal junction adenocarcinoma (GATSBY): An international randomised, open-label, adaptive, phase 2/3 study. Lancet Oncol..

[110] Ogitani Y., Aida T., Hagihara K., Yamaguchi J., Ishii C., Harada N., Soma M., Okamoto H., Oitate M., Arakawa S. (2016). DS-8201a, A Novel HER2-Targeting ADC with a Novel DNA Topoisomerase I Inhibitor, Demonstrates a Promising Antitumor Efficacy with Differentiation from T-DM1. Clin. Cancer Res..

[111] Modi S., Saura C., Yamashita T., Park Y.H., Kim S.B., Tamura K., Andre F., Iwata H., Ito Y., Tsurutani J. (2020). Trastuzumab Deruxtecan in Previously Treated HER2-Positive Breast Cancer. N. Engl. J. Med..

[112] Hurvitz S.A., Hegg R., Chung W.P., Im S.A., Jacot W., Ganju V., Chiu J.W.Y., Xu B., Hamilton E., Madhusudan S. (2023). Trastuzumab deruxtecan versus trastuzumab emtansine in patients with HER2-positive metastatic breast cancer: Updated results from DESTINY-Breast03, a randomised, open-label, phase 3 trial. Lancet.

[113] Deeks E.D. (2021). Disitamab Vedotin: First Approval. Drugs.

[114] Chen Z., Yuan J., Xu Y., Zhang C., Li Z., Gong J., Li Y., Shen L., Gao J. (2021). From AVATAR Mice to Patients: RC48-ADC Exerted Promising Efficacy in Advanced Gastric Cancer With HER2 Expression. Front. Pharmacol..

[115] Xu Y., Wang Y., Gong J., Zhang X., Peng Z., Sheng X., Mao C., Fan Q., Bai Y., Ba Y. (2021). Phase I study of the recombinant humanized anti-HER2 monoclonal antibody-MMAE conjugate RC48-ADC in patients with HER2-positive advanced solid tumors. Gastric Cancer.

[116] Sheng X., Yan X., Wang L., Shi Y., Yao X., Luo H., Shi B., Liu J., He Z., Yu G. (2021). Open-label, Multicenter, Phase II Study of RC48-ADC, a HER2-Targeting Antibody-Drug Conjugate, in Patients with Locally Advanced or Metastatic Urothelial Carcinoma. Clin. Cancer Res..

[117] Sheng X.A., He Z.S., Han W.Q., Zhou A.P., Luo H., Shi Y.X., Hu C.L., Liu Z.L., Guo H.Q., Yao X. (2021). An open-label, single-arm, multicenter, phase II study of RC48-ADC to evaluate the efficacy and safety of subjects with HER2 overexpressing locally advanced or metastatic urothelial cancer (RC48-C009). J. Clin. Oncol..

[118] Elgersma R.C., Coumans R.G., Huijbregts T., Menge W.M., Joosten J.A., Spijker H.J., de Groot F.M., van der Lee M.M., Ubink R., van den Dobbelsteen D.J. (2015). Design, Synthesis, and Evaluation of Linker-Duocarmycin Payloads: Toward Selection of HER2-Targeting Antibody-Drug Conjugate SYD985. Mol. Pharm..

[119] Black J., Menderes G., Bellone S., Schwab C.L., Bonazzoli E., Ferrari F., Predolini F., De Haydu C., Cocco E., Buza N. (2016). SYD985, a Novel Duocarmycin-Based HER2-Targeting Antibody-Drug Conjugate, Shows Antitumor Activity in Uterine Serous Carcinoma with HER2/Neu Expression. Mol. Cancer Ther..

[120] Menderes G., Bonazzoli E., Bellone S., Black J., Altwerger G., Masserdotti A., Pettinella F., Zammataro L., Buza N., Hui P. (2017). SYD985, a novel duocarmycin-based HER2-targeting antibody-drug conjugate, shows promising antitumor activity in epithelial ovarian carcinoma with HER2/Neu expression. Gynecol. Oncol..

[121] Menderes G., Bonazzoli E., Bellone S., Black J., Predolini F., Pettinella F., Masserdotti A., Zammataro L., Altwerger G., Buza N. (2017). SYD985, a Novel Duocarmycin-Based HER2-Targeting Antibody-Drug Conjugate, Shows Antitumor Activity in Uterine and Ovarian Carcinosarcoma with HER2/Neu Expression. Clin. Cancer Res..

[122] van der Lee M.M., Groothuis P.G., Ubink R., van der Vleuten M.A., van Achterberg T.A., Loosveld E.M., Damming D., Jacobs D.C., Rouwette M., Egging D.F. (2015). The Preclinical Profile of the Duocarmycin-Based HER2-Targeting ADC SYD985 Predicts for Clinical Benefit in Low HER2-Expressing Breast Cancers. Mol. Cancer Ther..

[123] Banerji U., van Herpen C.M.L., Saura C., Thistlethwaite F., Lord S., Moreno V., Macpherson I.R., Boni V., Rolfo C., de Vries E.G.E. (2019). Trastuzumab duocarmazine in locally advanced and metastatic solid tumours and HER2-expressing breast cancer: A phase 1 dose-escalation and dose-expansion study. Lancet Oncol..

[124] Zhang J., Liu R., Gao S., Li W., Chen Y., Meng Y., Liu C., Jin W., Wu J., Wang Y. (2023). Phase I study of A166, an antibody–drug conjugate in advanced HER2-expressing solid tumours. NPJ Breast Cancer.

[125] Humphreys R.C., Kirtely J., Hewit A., Biroc S., Knudsen N., Skidmore L., Wahl A. (2015). Site specific conjugation of ARX-788, an antibody drug conjugate (ADC) targeting HER2, generates a potent and stable targeted therapeutic for multiple cancers. Cancer Res..

[126] Zhang J., Ji D., Shen W., Xiao Q., Gu Y., O’Shaughnessy J., Hu X. (2022). Phase I Trial of a Novel Anti-HER2 Antibody-Drug Conjugate, ARX788, for the Treatment of HER2-Positive Metastatic Breast Cancer. Clin. Cancer Res..

[127] Zhang Y., Qiu M.Z., Wang J.F., Zhang Y.Q., Shen A., Yuan X.L., Zhang T., Wei X.L., Zhao H.Y., Wang D.S. (2022). Phase 1 multicenter, dose-expansion study of ARX788 as monotherapy in HER2-positive advanced gastric and gastroesophageal junction adenocarcinoma. Cell Rep. Med..

[128] Park Y.H., Ahn H.K., Kim J.Y., Ahn J.S., Im Y.H., Kim S.H., Lee S., Chung H.S., Park S.J. (2020). First-in-human phase I study of ALT-P7, a HER2-targeting antibody-drug conjugate in patients with HER2-positive advanced breast cancer. J. Clin. Oncol..

[129] Sharma M., Dumbrava E.I., Carvajal R., Catenacci D., Emens L., Hanna G., Juric D., Kang Y.K., Lee J., Lee K.W. (2020). Phase 1/2 Study of Novel Her2-Targeting, Tlr7/8 Immune-Stimulating Antibody Conjugate (Isac) Bdc-1001 with or without Immune Checkpoint Inhibitor in Patients with Advanced Her2-Expressing Solid Tumors. J. Immunother. Cancer.

[130] Graziani E.I., Sung M., Ma D.S., Narayanan B., Marquette K., Puthenveetil S., Tumey L.N., Bikker J., Casavant J., Bennett E.M. (2020). PF-06804103, A Site-specific Anti-HER2 Antibody-Drug Conjugate for the Treatment of HER2-expressing Breast, Gastric, and Lung Cancers. Mol. Cancer Ther..

[131] Meric-Bernstam F., Calvo E., Lee K.S., Moreno V., Park Y.H., Rha S.Y., Chalasani P., Zhong W., Zhou L., Pirie-Shepherd S. (2023). Safety and Tolerability of a Novel Anti-HER2 Antibody-Drug Conjugate (PF-06804103) in Patients With HER2-Expressing Solid Tumors: A Phase 1 Dose Escalation Study. Mol. Cancer Ther..

[132] Guo Y., Xue J., Peng W., Xue L., Ge X., Zhao W., Tang W., Nian W., Li Q., Zhang S. (2021). First-in-human, phase I dose escalation and expansion study of anti-HER2 ADC MRG002 in patients with HER2 positive solid tumors. Ann. Oncol..

[133] Hamblett K.J., Barnscher S.D., Davies R.H., Hammond P.W., Hernandez A., Wickman G.R., Fung V.K., Ding T., Garnett G., Galey A.S. (2019). ZW49, a HER2 targeted biparatopic antibody drug conjugate for the treatment of HER2 expressing cancers. Cancer Res..

[134] Jhaveri K., Han H., Dotan E., Oh D.Y., Ferrario C., Tolcher A., Lee K.W., Liao C.Y., Kang Y.K., Kim Y.H. (2022). Preliminary results from a phase I study using the bispecific, human epidermal growth factor 2 (HER2)-targeting antibody-drug conjugate (ADC) zanidatamab zovodotin (ZW49) in solid cancers. Ann. Oncol..

[135] Odegard V.H., Moyes K., Childs M., Brevik J., Winship D., Brender T., Metz H., Chang J.R., Adamo J., Setter B. (2020). Preclinical studies support the development of SBT6050, an anti-HER2 antibody conjugated to a potent TLR8 agonist, for treatment of moderate and high HER2-expressing tumors that lack pre-existing T cell infiltrate. Cancer Res..

[136] Oh D.Y., Bang Y.J. (2020). HER2-targeted therapies—A role beyond breast cancer. Nat. Rev. Clin. Oncol..

